# Supplementary material for: Comparison of antibody titres between intradermal and intramuscular rabies vaccination using inactivated vaccine in cattle in Bhutan
Source: PLoS One. 2019 Jun 10;14(6):e0209946. doi: 10.1371/journal.pone.0209946 (PMC6557474; doi:10.1371/journal.pone.0209946)

**S1. Fig. Pairwise comparisons of the effect of time since vaccination on proportion of vaccinated cattle with rabies VNA titres ≥0.24 IU/mL.**
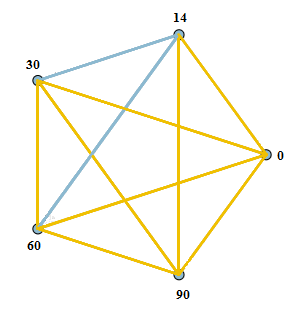

Supplement: S1 Fig — (DOCX) [file pone.0209946.s002.docx]
