# Supplementary material for: Comparison of antibody titres between intradermal and intramuscular rabies vaccination using inactivated vaccine in cattle in Bhutan
Source: PLoS One. 2019 Jun 10;14(6):e0209946. doi: 10.1371/journal.pone.0209946 (PMC6557474; doi:10.1371/journal.pone.0209946)
Supplement: S1 Table — (DOCX) [file pone.0209946.s003.docx]

|  |  | Robust |  |  |  |  |
| --- | --- | --- | --- | --- | --- | --- |
| **y** | **Coef.** | **Std. Err.** | **z** | **P>z** | **[95% Conf.** | **Interval]** |
|  |  |  |  |  |  |  |
| day |  |  |  |  |  |  |
| 14 | 1.726604 | 0.162088 | 10.65 | 0 | 1.408917 | 2.044291 |
| 30 | 1.950411 | 0.198026 | 9.85 | 0 | 1.562288 | 2.338535 |
| 60 | 1.32105 | 0.183439 | 7.2 | 0 | 0.961517 | 1.680583 |
| 90 | 0.799495 | 0.184037 | 4.34 | 0 | 0.438789 | 1.1602 |
|  |  |  |  |  |  |  |
| 1.vaccination | 0.428376 | 0.389214 | 1.1 | 0.271 | -0.33447 | 1.191221 |
|  |  |  |  |  |  |  |
| age |  |  |  |  |  |  |
| 1 | 0.597787 | 0.396487 | 1.51 | 0.132 | -0.17931 | 1.374886 |
| 2 | 0.910639 | 0.387973 | 2.35 | 0.019 | 0.150226 | 1.671053 |
|  |  |  |  |  |  |  |
| vaccination#age | |  |  |  |  |  |
| 1 1 | -0.9302 | 0.475015 | -1.96 | 0.05 | -1.86122 | 0.000808 |
| 1 2 | -1.3988 | 0.453407 | -3.09 | 0.002 | -2.28746 | -0.51014 |
|  |  |  |  |  |  |  |
| _cons | -2.54445 | 0.384736 | -6.61 | 0 | -3.29852 | -1.79038 |

**S1 Table. Generalised estimating equation model outputs**
